# Jingzhaotoxin-X, a gating modifier of Kv4.2 and Kv4.3 potassium channels purified from the venom of the Chinese tarantula *Chilobrachys jingzhao*

**DOI:** 10.1590/1678-9199-JVATITD-2019-0043

**Published:** 2020-05-29

**Authors:** Meichun Deng, Liping Jiang, Xuan Luo, Huai Tao, Songping Liang

**Affiliations:** 1 Department of Biochemistry and Molecular Biology, School of Life Sciences, Central South University, Changsha, Hunan 410013, China.; 2 Department of Parasitology, Xiangya School of Medicine, Central South University, Changsha 410013, China.; 3 The National and Local Joint Engineering Laboratory of Animal Peptide Drug Development, College of Life Sciences, Hunan Normal University, Changsha, 410081, China.; 4 Department of Biochemistry and Molecular Biology, Hunan University of Chinese Medicine, Changsha 410208, Hunan, China.

**Keywords:** Tarantula toxin, Potassium channels, Kv4, Patch-clamp, Pain

## Abstract

**Background:**

The tarantula *Chilobrachys jingzhao* is one of the largest venomous spiders in China. In previous studies, we purified and characterized at least eight peptides from *C. jingzhao* venom. In this report, we describe the purification and characterization of Jingzhaotoxin-X (JZTX-X), which selectively blocks Kv4.2 and Kv4.3 potassium channels.

**Methods:**

JZTX-X was purified using a combination of cation-exchange HPLC and reverse-phase HPLC. The amino-acid sequence was determined by automated Edman degradation and confirmed by mass spectrometry (MS). Voltage-gated ion channel currents were recorded in HEK293t cells transiently transfected with a variety of ion channel constructs. In addition, the hyperalgesic activity of JZTX-X and the toxin´s effect on motor function were assessed in mice.

**Results:**

JZTX-X contained 31 amino acids, with six cysteine residues that formed three disulfide bonds within an inhibitory cysteine knot (ICK) topology. In whole-cell voltage-clamp experiments, JZTX-X inhibited Kv4.2 and Kv4.3 potassium channels in a concentration- and voltage-dependent manner, without affecting other ion channels (Kv1.1, 1.2, 1.3, 2.1, delayed rectifier potassium channels, high- and low-voltage-activated Ca2+ channels, and voltage-gated sodium channels Nav1.5 and 1.7). JZTX-X also shifted the voltage-dependent channel activation to more depolarized potentials, whereas extreme depolarization caused reversible toxin binding to Kv4.2 channels. JZTX-X shifted the Kv4.2 and Kv4.3 activities towards a resting state, since at the resting potential the toxin completely inhibited the channels, even in the absence of an applied physical stimulus. Intrathecal or intraplantar injection of JZTX-X caused a long-lasting decrease in the mechanical nociceptive threshold (hyperalgesia) but had no effect on motor function as assessed in the rotarod test.

**Conclusions:**

JZTX-X selectively suppresses Kv4.2 and Kv4.3 potassium channel activity in a concentration- and voltage-dependent manner and causes long-lasting mechanical hyperalgesia.

## Introduction

The voltage-gated potassium (Kv) channels represent the diverse and extensively distributed proteins that are critically involved in maintaining the resting membrane potential, repolarization of action potential, as well as signal transduction [1]. Among the various Kv channels, the Shal-type (Kv4) family is characterized by fast inactivation and activation, which consists of 3 different members (Kv4.1-4.3) encoded via different genes (namely, KCND1-3 within human beings) and expressing the transient potassium currents within both the heart and nervous system [2]. Kv4 channels within the nervous system limit the action potential back-propagation, assist in establishing the slowly repeated spike firing, and facilitate the signal amplification and spike repolarization [3, 4]. In the heart, Kv4 channels are involved in numerous physiological processes, such as synaptic transmission, membrane excitability, and repolarization of cardiac myocytes [5, 6].

The tarantula *Chilobrachys jingzhao* is one of the largest venomous spiders in the south of China. In previous studies, we purified and characterized at least eight peptides from *C. jingzhao* venom (as shown in Table 1). Peptide toxins purified from spider venom provide crucial approaches to investigating Kv4 channels. Typically, Jingzhaotoxin-V, phrixotoxins (PaTx1-2), heteropodatoxins (HpTx1-3), and SNX-482 are the short peptides consisting of 29-35 amino-acid residues with three disulfide bonds, which inhibit either Kv4.3 or Kv4.2, or both of these channels [14, 19-21]. Jingzhaotoxin-XII, which is separated from the venom of the Chinese tarantula *C. jingzhao*, serves as a specific Kv4.1 channel-gating modifier [17]. HmTx1-2 and ScTx1, are 34- to 38-amino acid peptides that belong to the structural family of inhibitor cystine knot motif reticulated by three disulfide bridges, exhibit different affinities to Kv2.1 and Kv4 channels [22]. These toxins are routinely used as pharmacological approaches to physiologically investigating various subunits of potassium channels in potential therapeutics and cellular physiology[23].

**Table 1.** Characterization of the main peptides from *C. jingzhao* venom

Toxin name	Abbreviation	Molecular mass (Da)	Amino-acid sequence	Main pharmacological activity	Reference
Jingzhaotoxin-I	JZTX-I	3675	ACGQFWWKCGEGKPPCCANFACKIGLYLCIWSP	Inhibits Nav1.5 channels inactivation and Kv2.1 channels	[7-9]
Jingzhaotoxin-II	JZTX-II	3561	GCGTMWSPCSTEKPCCDNFSCQPAIKWCIWSP	Inhibits inactivation of both rat cardiac Nav channels and Nav1.5 channels	[10]
Jingzhaotoxin-III	JZTX-III	3918	DGECGGFWWKCGRGKPPCCKGYACSKTWGWCAVEAP	Inhibits Nav1.5 and Kv2.1 channels	[11, 12]
Jingzhaotoxin-IV	JZTX-IV	3775	ECTKFLGGCSEDSECCPHLGCKDAVLYYCAWDGT	Modifies the activation and inactivation of Nav channels	[13]
Jingzhaotoxin-V	JZTX-V	3605	YCQKWMWTCDSKRACCEGLRCKLWCRKII	Inhibits both Nav and Kv4 channels	[14]
Jingzhaotoxin-XI	JZTX-XI	3727	ECRKMFGGCSVDSDCCAHLGCKPTLKYCAWDGTF	Inhibits Nav1.5 inactivation and Kv2.1 channels	[15, 16]
Jingzhaotoxin-XII	JZTX-XII	3665	YCQKWMWTCDSERKCCEGYVCELWCKYNGG	Inhibits Kv4.1 channels	[17]
Jingzhaotoxin-XIII	JZTX-XIII	4123	ECRWLFGGCEKDASDCCEHLGCRRAKPSWCGWDFTF	Modifies Kv channel gating	[18]

In this study, we identify the properties of Jingzhaotoxin-X (JZTX-X), a new neurotoxin obtained from the Chinese tarantula *Chilobrachys jingzhao* venom. As a polypeptide containing 31 residues, this toxin has 3 disulfide bonds, with a non-amidated C-terminal residue. According to our results, JZTX-X potently suppresses Kv4.2 and Kv4.3 channels, without affecting other ion channels. JZTX-X also shifts the voltage-dependent channel activation to more depolarized potentials, and captures the closed-state voltage sensor. JZTX-X serves as a valuable approach to investigating Kv4.2 and Kv4.3 channels for their gating mechanisms.

## Material and methods

### Purification of toxin

JZTX-X was purified from *C. jingzhao* venom using a combination of cation-exchange high-performance liquid chromatography (HPLC) and RP-HPLC [24]. The toxin purity used in this study was 98%, as detected by the matrix-assisted laser desorption/ionization time-of-flight (MALDI-TOF) analysis and HPLC. In each step, the Kv4 channel-modulating activity was detected in the fractions.

### Plasmids of Kv4.2 and Kv4.3 channels and transient transfection

Rat cDNA genes encoding Kv4.2 (Accession: NM_031730.2) and Kv4.3 (Accession: AB003587.1) were subcloned to vector pcDNA3.1. In addition, human cDNA genes encoding Nav1.7 (Accession: DQ857292.1) and Nav1.5 (Accession: AB158469.2) were subcloned to vectors pcDNA3.1-mod and pcDNA3.1, separately. Then, Kv4.2, Kv4.3, Nav1.5 and Nav1.7 constructs were transiently transfected into the human embryonic kidney 293t (HEK293t) cells using lipofectamine 2000 (Invitrogen, USA) in accordance with manufacturer protocols. Thereafter, HEK293 cells were inoculated within Dulbecco’s modified Eagle’s medium (DMEM) containing 10% fetal bovine serum (FBS) at 5% CO_2_ and 37°C. The Kv4.3 channel and the Kv4.2 counterpart were then transfected in the absence of β subunit. Subsequently, cells were incubated with a DNA-lipofectamine mixture (one reporter plasmid for green fluorescence protein and channel constructs) for 4-6 h, and were washed with the fresh medium. Finally, cells that displayed green fluorescent protein fluorescence were screened for whole-cell patch-clamp assays after transfection for 36-72 h. 

### Whole-cell patch-clamp assays

An EPC-10 patch-clamp amplifier (HEKA, Germany) was employed to record the potassium currents at the configuration of whole-cell patch-clamp under an ambient temperature of 22-25°C. The resistance was 2.0-3.0 MΩ when the recording pipette contained a full volume of internal solution (supplemented with 140 mM KCl, 10 mM HEPES, 2.5 mM MgCl_2_, 5 mM ATP and 11 mM EGTA). KOH was utilized to adjust the pH to 7.2, while sucrose was employed to adjust the osmolarity to 310 mOsm/L. The culture medium was replaced with an external solution containing 150 mM NaCl, 2.5 mM CaCl_2_, 10 mM HEPES, 2 mM MgCl_2_, 5 mM KCl, and 10 mM D-glucose. Similarly, sucrose and NaOH were applied to adjust the osmolarity to 330 mOsm/L and pH to 7.4. Thereafter, currents were observed for 10 min, and cells with noticeably reduced currents in the abovementioned period were removed. The series resistance was maintained at about 5 MΩ, with 65-70% compensation. Finally, the leakage currents and linear capacitance were subtracted digitally according to the P/4 protocol.

### Microelectrode voltage-clamp assays

Briefly, plasmids were linearized, and transcription was carried out according to the standardized protocol. Afterwards, the capped cRNAs that encoded the related ion channels were prepared [25]. To conduct transcription *in vitro*, firstly, NotL was utilized to linearize plasmids pCI that contained both Kv1.1 (Accession: NM_010595.3) [26] and Kv2.1 (Accession: AF026005.1) [27], whereas SphI was adopted to linearize plasmid PcDNA3 containing Kv1.2 (Accession: NM_012970.3) gene [28]. NotI was adopted to linearize plasmid pCI-neo possessing Kv1.3 (Accession: BC035059.1) gene [29]. Pst I or SmaI was utilized to linearize plasmid PcDNA3.1 that possessed Kv4.3 or Kv4.2 gene[30]. Thereafter, the linearized plasmids were used as the templates to synthesize cRNAs *in vitro* using the large-scale T7 mMESSAGE mMACHINE transcription kit (Ambion, USA).

A mature female X. laevis was placed on the ice for anesthesia, and then the *Xenopus laevis* oocytes at stage IV-VI were extracted. Subsequently, those extracted oocytes were treated by collagenase (1 mg/mL) contained within the calcium-depleted ND96 solution (containing 96 mM NaCl, 10 mM HEPES, 1 mM MgCl_2_, and 2 mM KCl) for defolliculation. At 2 h and 24 h after defolliculation, the oocytes were injected with 41 nl cRNA (100-500 ng/ll) via a microprocessor-controlled nanoliter injector (WPI, USA). Subsequently, the oocytes were incubated within the OR_2_ solution (pH = 7.5) for 1-4 days at 18°C. On the other hand, the OR_2_ solution was supplemented with 82.5 mM NaCl, 1 mM CaCl2, 2.5 mM KCl, 1 mM Na2HPO4, 5 mM HEPES, 1 mM MgCl2, and gentamycin sulfate (50 mg/L) for simple incubation.

The two-microelectrode voltage-clamp (TURBO TEC-03X, NPI Electronic, Germany) was adopted to record the whole-cell currents in those oocytes. 3M KCl was employed to fill the current and voltage electrodes (0.1-1 MΩ), respectively. The recording chamber was perfused with an extracellular solution (supplemented with 50 mM KCl, 1 mM MgCl_2_, 50 mM NaCl, 0.3 mM CaCl_2_, and 5 mM HEPES, and NaOH was applied to adjust the pH to 7.5. Later, oocytes were investigated within this recording chamber. As for currents, the records were extracted at an interval of 0.5 ms following low-pass filtering at 2 kHz. The linear capacity components, together with the leak currents, were not subtracted. Each assay was carried out under ambient temperature (19-23°C).

### Molecular Modeling

The 3D JZTX-X model was acquired through homology modeling based on a ω-Grammotoxin SIA structure (PDB code: 1KOZ) determined by the NMR technique [31]. Typically, the ω-Grammotoxin SIA structure was utilized as the template due to its high primary sequence similarity and identical cysteine positions. Then, samples were submitted to the ESyPred3D web server for molecular modeling [32]. The software DS ViewerPro (Accelrys, America) was employed for visualizing structure.

### Tests for nociceptive behaviors

In the present study, male C57BL/6 mice weighing 20-25 g were utilized. This study was performed following the Guide for the Care and Use of Laboratory Animals released by the United States National Institutes of Health (US NIH). Our study protocol was approved by the Animal Experiments Ethics Committee at Central Southern University (No. 2018-2-6). Each operation was performed under isoflurane-induced anesthesia to reduce pain and minimize animal suffering. Lumbar puncture was also conducted for intrathecal injection, as described previously [33]. Next, the effect of JZTX-X on nociception was evaluated by assessing the behaviors during the mechanical hyperalgesia process. The JZTX-X-induced mechanical hyperalgesia was investigated by two experiments via various administration routes, including intrathecal and intraplantar injections. Then, animals were challenged via intrathecal (10 µL) or intraplantar (20 µL) injection of JZTX-X (40 nmol/kg). The control group was given a physiological solution, which contained 0.9% NaCl, 10 µL intraplantar injection and 20 µL intrathecal injection.

Mechanical hyperalgesia was measured according to the previously depicted “up-down” paradigm [25]. Individual mice were allowed to acclimate for at least 30 min on the mesh floor in suspended chambers. After acclimatization, various calibrated von Frey filaments (Stoelting, Wood Dale, IL) were applied to all animals, which were vertical to the hind paw plantar surfaces becausethere was enough force for bending those filaments for 6 s. Later, the animal responses were recorded. The positive response was defined as paw flinching or brisk withdrawal. When there was no response, force filament at a greater level was applied. Accordingly, force ligament at a lower level was applied when there was no response. The “up-down” approach was adopted to calculate the 50% likelihood where one withdrawal response was produced by the simple stimulus.

A rotarod accelerator treadmill (Med Associates Inc., St. Albans, VT) was utilized for rotarod performance tests, so as to determine rat motor function as previously described [25]. Motor learning was also carried out by rotarod tests for evaluating the mice’s capacity to stay on the accelerating rotating drum. In JZTX-X- and saline- groups, all animals were acclimated for 30 min within the testing room, and placed for 300 s onto the rotarod that accelerated from 4 to 40 rpm. In addition, the latency time for animals falling from the rod was calculated before and after toxin injection, from the beginning of acceleration to falling onto the drum, and the maximum cutoff time was 300 s.

### Data analysis

All experimental results were obtained and examined using Sigmaplot (Sigma, USA) and Pulse-Pulsefit8.0 (HEKA, Germany), and presented as mean ± standard error (S.E.), in which *n* represented the independent experiment number. In addition, the concentration-response curves for determining IC_50_ values were fitted based on the Hill Equation (1): y = 1-(1-*f*_max_)/(1 + ([x] /IC_50_)^nH^), in which *x* stands for toxin dose, *n*_*H*_ represents the Hill Coefficient (slope parameter), whereas *IC*_*50*_ is indicative of the median inhibitory dose resulting in membrane current block or lethality. 

On-rates were determined by fitting time course data with a single exponential decay function (2): y = Ae^-kx^ + C, where *x* is the time, *A* is the normalized current value before the application of toxin (usually 1.0), and *C* is the final normalized current value following the block by the toxin. The on-rate (τ_on_) was determined from the inverse of the rate constant *k*.

Off-rates were determined by fitting time course data with a double exponential association function (3): y = A(1-e^-kx^) + B(1-e^-fx^) + C, where *x* is the time, *A* or *B* is the normalized current value after washout of the toxin (usually 1.0 if complete washout occurred), and *C* is the normalized current value before the washout of the toxin. The off-rate (τ_off_) was determined from the inverse of the rate constant *k* or *f*.

As for kinetic calculations, curves as a function of peak currents vs. time were plotted from the toxin wash-out or wash-in moments. The software Sigmaplot 9.0 (Sigma, USA) was utilized to fit data to the first-order exponential equation. Thereafter, kinetic constants were computed for toxin dissociation (*k*_*off*_ ) and channel modification (*k*_*mod*_ ) through the τ inverse, so as to fit the toxin wash-out and wash-in, respectively. The following equation was adopted to determine the toxin association rate, which was k_on_ = k_mod_ - k_off_ / [toxin]. The equation K_d_ =k_off_ / k_on_ was adopted to determine the dissociation constant.

## Results

### Purification, analysis of amino-acid sequences, and structure model of JZTX-X

**Figure 1.**
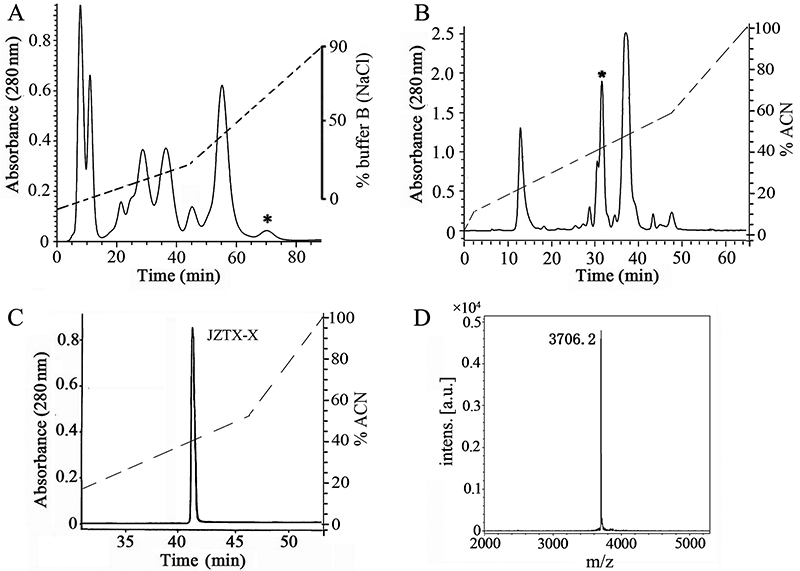
Purification of JZTX-X. **(** A **)** Cation-exchange HPLC elution profile of *C. jingzhao* venom (10 mg): Peptides and proteins were eluted with a non-linear gradient (0-90%, dashed line) of 1 M NaCl in 0.02 M sodium phosphate buffer (pH 6.25) at a flow rate of 3 ml/min and the elution profile was monitored at 280 nm. **(** B **)** RP-HPLC of the active fraction from the previous step: The C18 column was pre-equilibrated with 0.1% trifluoroacetic acid (TFA) and the peptides were eluted with a non-linear gradient (0-100%) of acetonitrile in 0.1% TFA. The asterisk (*) in (A) and (B) indicates the active peak. **(** C **)** Analytical RP-HPLC (C18 column) of purified toxin (JZTX-X): The column was pre-equilibrated with 0.1% TFA and the toxin was eluted with a linear gradient (0-100%) of acetonitrile in 0.1% TFA. The flow rate in (B) and (C) was 3 ml/min and the elution profile was monitored at 280 nm in both cases. **(** D **)** Confirmation of the purity of JZTX-X and determination of the molecular mass of the intact toxin by MALDI-TOF mass spectrometry.

JZTX-X was purified from *C. jingzhao* venom using a combination of cation-exchange HPLC and RP-HPLC. For each chromatographic run, 10 mg of lyophilized venom dissolved in 2 ml of distilled water was used for cation exchange HPLC (Fig. 1A), with a total of 30 runs done. The active peak (detected based on activity towards Kv4.2 channels) was then chromatographed on a C18 RP-HPLC column pre-equilibrated with 0.1% trifluoroacetic acid (TFA) and eluted with a gradient of acetonitrile in 0.1% TFA (Fig. 1B). The purity of the naturally occurring toxin was over 98%, as determined by and RP-HPLC and MALDI-TOF mass spectrometry (Figs. 1C and 1D). The yield of JZTX-X by this protocol was ~1% of the dry venom weight.

**Figure 2.**
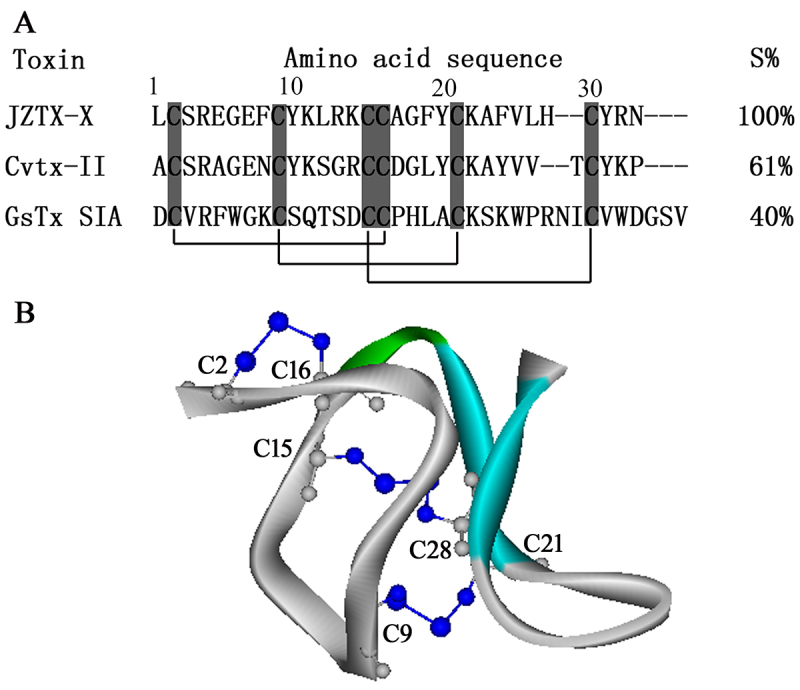
Sequence alignment and structure models of JZTX-X: **(** A **)** JZTX-X sequence was aligned with specific toxins obtained from various spider venoms, including Cvtx-II from *Coremiocnemis validus* [34] and ω-grammotoxin SIA from *Grammostola spatulata* venom [35]. The similarity percentage was presented relative to JZTX-X. **(** B **)** The schematic model of JZTX-X displaying the position of secondary structure: JZTX-X structure models were acquired based on homology modeling according to ω-grammotoxin SIA structure detected by NMR (PDB code: 1KOZ). The schematic models were established by DS ViewerPro. The β-sheet is shown in *blue*, and the three disulfide bonds (including Cys15-Cys28, Cys9-Cys21, and Cys2-Cys16) are indicated.

The complete amino-acid sequence of JZTX-X was determined by automated Edman degradation and verified by mass spectrometry. The results suggested that the active peak was a 31-amino-acid polypeptide including six cysteine residues at positions 2, 9, 15, 16, 21 and 28 (Fig. 2). The calculated linear reduction of JZTX-X mass (3712.4 Da) was 6 Da higher than its monoisotopic molecular mass (M+H)^+^ as judged by mass spectrometry (3706.2 Da), suggesting that all 6 cysteine residues contained within JZTX-X participated in the formation of 3 intramolecular disulfide bridges. Moreover, putative monoisotopic molecular mass (M+H)^+^ of unamiable toxin (3,706.1 Da) was consistent with the experimental value for natural JZTX-X, which indicated that the natural JZTX-X, namely huwentoxin-XVI (HWTX-XVI), was unamiable at the C-terminal in post-translational regulation [25].

Additionally, alignment with other spider toxins suggested that JZTX-X shared significant similarity with covalitoxin-II (Cvtx-II, 61%) derived from *Coremiocnemis validus* (Singapore tarantula) venom [34] and ω-grammotoxin SIA (GsTx SIA, 40%), the blocker of calcium channel obtained based on venom from the Chilean tarantula *Grammostola spatulata* [35]. A total of 6 cysteine residues were highly conserved among each spider toxin at specific locations. The JZTX-X analogues are suggested in experiments to be frequently involved in the formation of cysteine connectivity (C3-C6, C2-C5, and C1-C4) that occurs in ICK peptides [36]. Therefore, we supposed that such identical cysteine connectivity was also adopted in JZTX-X (Fig. 2A). The JZTX-X sequence was further analyzed by the program Disulfind, a web-based approach for predicting and identifying the protein connectivity and the state of cysteine disulfide bonding (http://disulfind.dsi.unifi.it/) [37]. Based on our results, JZTX-X might be adherent to those canonical motifs of disulfide bridges. Also, the JZTX-X 3D model was established based on the GsTx SIA NMR structure. The JZTX-X molecular structure showed high similarity to the spider ICK motif toxin counterparts, which also possessed one β-turn, two β-strands, together with the abovementioned three turns stabilized by disulfide bonds. 

### JZTX-X effect on the voltage-gated potassium channels

**Figure 3.**
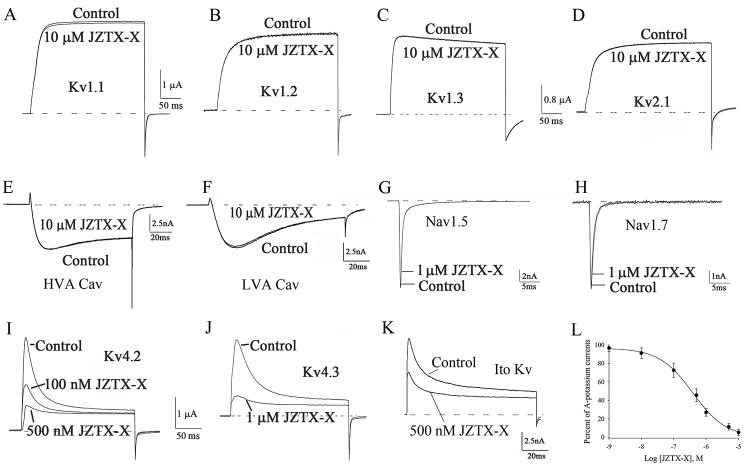
Effects of JZTX-X on the voltage-gated potassium channels in *X. laevis*oocytes: **(** A **-** D **)** 10 μM JZTX-X did not markedly affect the currents of the isoforms of delayed rectifier potassium channels Kv1.1 (A), Kv1.2 (B), Kv1.3 (C), or Kv2.1 (D) channels. 300-ms depolarization was applied to elicit the potassium currents to +20 mV from the -90 mV holding potential (Vh). **(** E **-** F **)** 10 μM JZTX-X apparently did not affect the currents of HVA Cav channels (E) or LVA Cav channels (F) from rat DRG neurons. **(** G **-** H **)** One μM JZTX-X did not affect the currents of Nav1.5 (G) or Nav1.7 channels (H). **(** I **)** 100 nM and 500 nM JZTX-X inhibited 57.8±4.6% and 83.8±5.1% of Kv4.2 currents, respectively. **(** J **)** 1 μM JZTX-X inhibited 72.4±4.8% of Kv4.3 currents. **(** K **)** 500 nM JZTX-X inhibited 54.4 ± 6.8% of Ito currents in rat DRG neurons. (L) Concentration-dependent inhibition of Ito channels by JZTX-X in rat DRG neurons.

Firstly, the effects of JZTX-X on the isoforms of potassium channels (including Kv1.1-1.3, Kv2.1, as well as Kv4.2-4.3) contained within the *X. laevis* oocytes were investigated through the microelectrode voltage-clamp approach. Then, 300ms depolarization was applied to elicit the outward potassium currents to +20 mV based on the -90 mV holding potential (Vh). Fig. 3 suggested that 4 isoforms (including Kv1.1-1.3, and Kv2.1) were JZTX-X-resistant, while Kv4.2 and Kv4.3 were toxin-sensitive. At the conditions of whole-cell voltage-clamp, JZTX-X at 10 μM had almost no effect on Kv1.1-1.3, Kv2.1, HVA or LVA Cav channels (less than 5% inhibition, n = 3, Fig. 3A, B, C, D, E, F). 1 μM JZTX-X also had a negligible effect on Nav1.5 or Nav1.7 channel (n = 3, Fig. 3G, H). However, the application of 0.1 or 0.5 μM JZTX-X rapidly inhibited the Kv4.2 currents on *X. laevis* oocytes (57.8±4.6% and 83.8±5.1%, respectively, Fig. 3I, n = 5). For Kv4.3 channels, those current amplitudes were depressed by 72.4±4.8% as the toxin content elevated to 1 μM (Fig. 3J, n = 5). We also determined the effects of JZTX-X on Ito potassium channels in rat DRG neurons; those current amplitudes were inhibited by 54.4±6.8% when 500 nM JZTX-X was applied (Fig. 3K, n = 5). When the dose-response data was fit to a Hill equation, which yielded the IC_50_ values of 386 nM for Ito potassium channels (Fig. 3L, n = 6).

**Figure 4.**
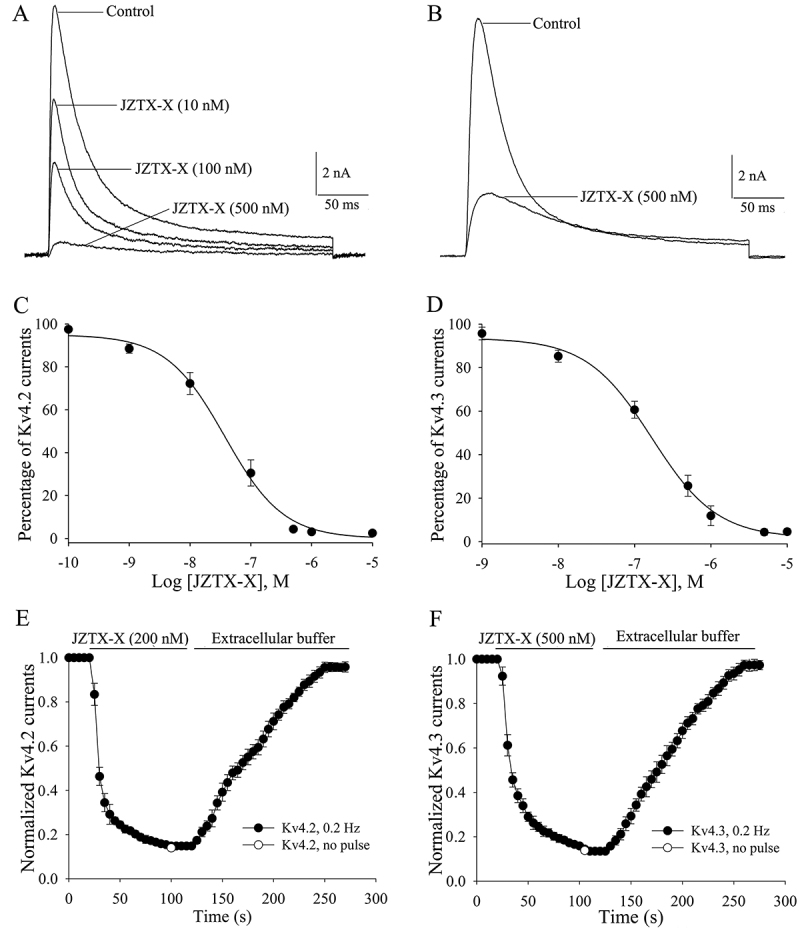
Effects of JZTX-X on Kv4.2 and Kv4.3 channels depending on dose and time: **(** A **,** B **)** original recording traces showed the effect of JZTX-X on Kv4.2 (A) and Kv4.3 (B) channels in HEK 293T cells. (C, D) Concentration-dependent suppression of Kv4.2 (C) and Kv4.3 (D) channels by JZTX-X in HEK 293T cells held at the voltage of −80 mV. All data (mean ± S.E.) were obtained from 5-8 independent experimental cells, and fitted based on Eq. (1) (“Materials and methods”). **(** E **,** F **)** The off- and on-rates of Kv 4.2 (E) or Kv4.3 (F) channels after 1 μM or 10 μM JZTX-X treatment, and were washed with a toxin-free external solution (*n* = 6): *No pulse* suggested no pulse applied in Kv 4.2 (E) or Kv4.3 (F) channels (open circle) during the initial 100 s after toxin treatment. All data were expressed as mean ± S.E.M. and fitted based on Eqs. (2) and (3).

Secondly, the effects of JZTX-X (1 nM-10 μM) on Kv4.2 and Kv4.3 channels were detected in HEK293t cells. Fig. 4A and 4B displayed the representative Kv4.2 and Kv4.3 currents recorded within the 300-ms voltage steps to +20 mV at -80 mV holding potential in the presence or absence of JZTX-X, respectively. Extracellular application of JZTX-X reduced the peak amplitudes for Kv4.2 and Kv4.3 currents in a time- and dose-dependent manner. The IC_50_ values of 68 nM JZTX-X for Kv4.2 channels (Fig. 4C, n = 7) and 210 nM JZTX-X for Kv4.3 channels were obtained from the concentration-response data and fitted to the Hill Equation (Fig. 4D, n = 8), with the Hill Coefficients of 0.91 and 1.17, separately. The Kv4.2 and Kv4.3 currents were rapidly inhibited under high JZTX-X dose, which was almost totally reversed (>95%) in 3 min following rinsing (n = 6, Fig. 4 E, F). Typically, those time courses (τ_on_) to inhibit the Kv4.2 and Kv4.3 currents were maximally fitted at the voltage of +20 mV through the singular exponential function, and the time constants were 12.6 s and 15.7 s (n = 6, Fig. 4E, F, Table 2), respectively. At the -80 mV resting potential and 0 pulsed applied during the first ~100 s following 200 nM or 500 nM JZTX-X treatment, the Kv4.2 and Kv4.3 channels were inhibited by approximately 95%, and the suppression was the same aswhen the depolarizing pulse was applied at 5 s intervals (n = 5, Fig. 4E, F).

**Table 2.** Interactions between JZTX-X and voltage-gated potassium channels

Channels	k_mod_ (s^-1^)	k_off_ (s^-1^)	k_on_ (10^5^M^-1^s^-1^)	k_i_ (μM)	Test conc. (μM)
Kv4.2 channel (n = 6)	0.089 ± 0.007	0.008 ± 0.0006	0.84 ± 0.005	0.095 ± 0.03	0.2
Kv4.3 channel (n = 8)	0.073 ± 0.006	0.005 ± 0.0004	0.068 ± 0.006	0.735 ± 0.05	0.5

### JZTX-X effects on activation kinetics of Kv4.2 and Kv4.3 channels

**Figure 5.**
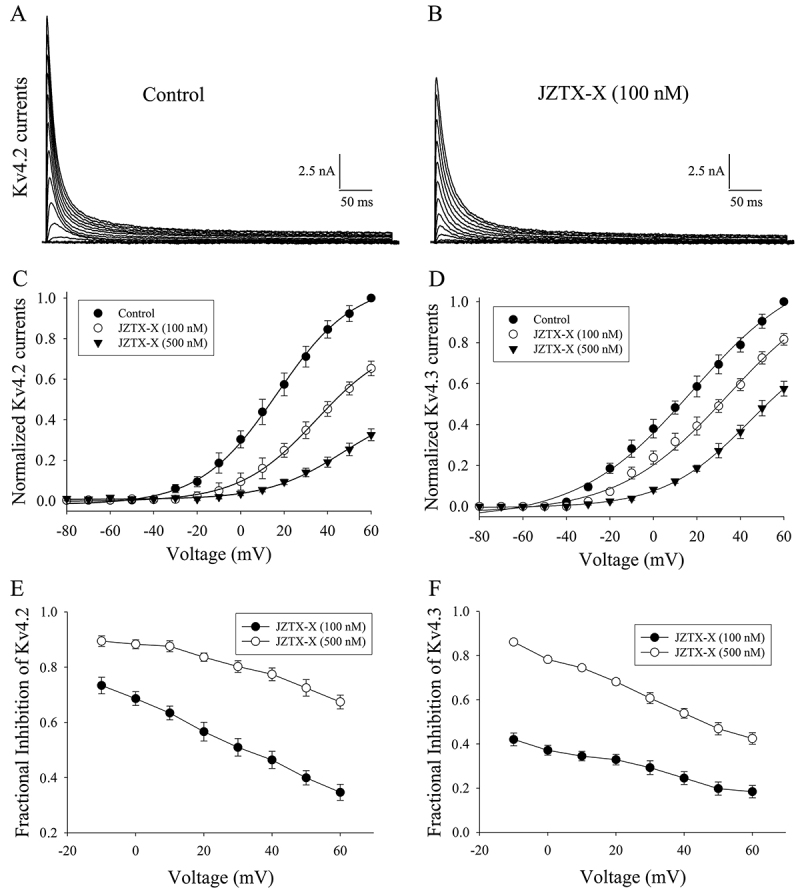
Effects of JZTX-X on I-V relationships in Kv4.2 and Kv4.3 channels: Each cell was held at the voltage of -80 mV, and the sodium current families were induced at the depolarization steps of 300 ms to different potentials (range, -80 mV to +60 mV at 10 mV increments). **(** A **)** and **(** B **)** showed the representative recording traces of I-V relation for the Kv4.2 currents before and after 100 nM JZTX-X treatment, respectively. **(** C **)** and **(** D **)** represented I-V relation for Kv4.2 (C) and Kv4.3 (D) currents before (filled circles) and after 100 nM (open circles) or 500 nM (filled inverted triangles) JZTX-X treatment, respectively. **(** E **)** and **(** F **)** represented the fractional suppression on Kv4.2 (E) and Kv4.3 (F) currents after 100 nM (filled circles) and 500 nM (open circles) JZTX-X treatment, respectively. All data were expressed as mean ± S.E.M (n = 5-7 cells).

The voltage-dependent inhibition of Kv4.2 and Kv4.3 channels by JZTX-X was determined by the depolarizing pulses to potentials in 10 mV steps from −80 to +60 mV at an interval of 10 s and the holding potential of -80 mV. Figs. 5A and =5B showed the relationship between current and voltage (I-V) for the Kv4.2 channels with and without 100 nM JZTX-X treatment. JZTX-X reduced the currents of Kv4.2 channels throughout the whole voltage range, and Kv4.2 was activated within this range. JZTX-X also exerted similar effects on the Kv4.3 channels (data not shown). For quantifying the voltage-depending inhibition of Kv4.2 and Kv4.3 channels, the relative current was drawn based on the membrane potential. Under control conditions, those threshold potentials to initially activate Kv4.2 and Kv4.3 currents were around -40 mV. The JZTX-X treatment at 500 nM inhibited the Kv4.2 and Kv4.3 currents, and then the I-V relation shifted to the right by about 30 mV (from -39.5 ± 1.9 mV to -11.3 ± 1.2 mV ) and 40 mV (from -39.8 ± 1.5 mV to -2.5 ± 0.9 mV ), separately (Fig. 5C and D). Additionally, the channel conductance also shifted rightward by around 30 mV (data not shown). Furthermore, the JZTX-X-induced inhibition of Kv4.2 and Kv4.3 currents was dependent on voltage, since the inhibition degree differed within the test potentials range (-10 to +60 mV) (Figs. 5 E and F). These results implied that JZTX-X might inhibit Kv4.2 channels through gating modification. 

### Strong depolarization induced the dissociation of JZTX-X from potassium channels

**Figure 6.**
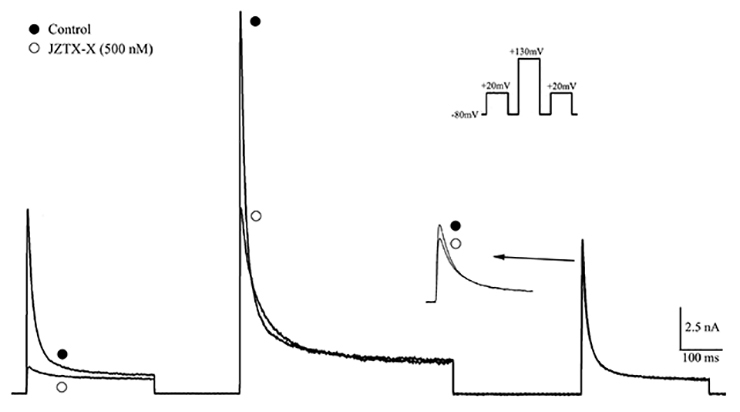
Potent depolarization dissociated the toxin-channel complex. The 300 ms test pulse at the voltage of +20 mV was utilized to induce the current traces. Prior to the application of test pulse, cells held at the voltage of -80 mV depolarized for 500 ms at the conditioning pulse of +130 mV and the holding potential of 50 ms (see inset). Following 500 nM JZTX-X perfusion, the test pulse-elicited current amplitude was nearly identical to the control after the 500 ms conditioning pulse.

Subsequently, we examined whether strong depolarization induced toxin dissociation from potassium channels. To address this question, the triple-pulse protocol was utilized, which used the test pulse of +20 mV after potent depolarization (+130 mV) to measure the available Kv4.2 currents (Fig. 6, inset). According to Figure 6, 500 nM JZTX-X almost completely inhibited the first pulse (+20 mV)-induced Kv4.2 currents (92.6 ± 3.2%) in HEK 293T cells. Furthermore, this toxin also affected the amplitude of outward Kv4.2 currents (46.3 ± 5.6%) by the second pulse (+130 mV). Typically, the amplitude of current acquired was nearly the same as that in controls induced by a third pulse (+20 mV), since the inhibition was only 17.5 ± 3.9% (Fig. 6, n = 7). Interestingly, such results revealed the effect of potent depolarization on reversing the inhibition on Kv4.2 potassium channel, which was ascribed to the toxin-channel complex dissociation.

### JZTX-X effects on Kv4.2 channel kinetics

**Figure 7.**
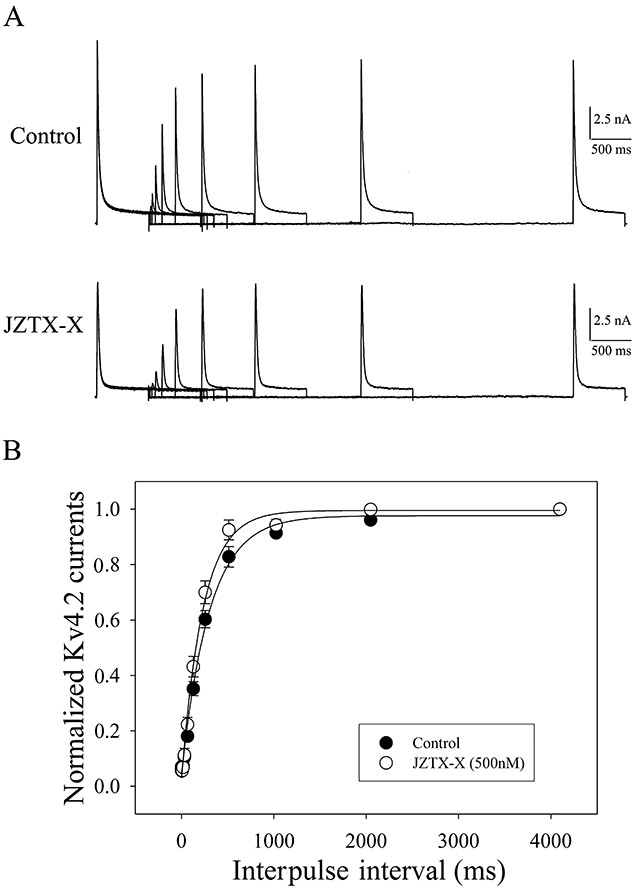
Effects of JZTX-X on the inactivation recovery time course of Kv4.2 channels. **(** A **)** The two-pulse protocol was applied to record the typical current traces with or without JZTX-X treatment. First of all, the initial 700 ms depolarization pre-pulse was applied at +20 mV from the holding potential of -80 mV, and then the second same pulse was applied at −80 mV when the interpulse intervals were between 0.5 ms and 4096 ms. Thereafter, those elicited peak currents in test pulses were determined and standardized based on the peak currents for conditioning pulses within the identical cell. The first record in each row is the ‘conditioning pulse’ employed to normalize the other records, and the last one is a ‘control’ utilized to confirm the full recovery of channel current. **(** B **)** The standardized recovery data were expressed as mean ± S.E. (n = 7), plotted as a function of inter-pulse interval, and fitted to one singular exponential function.

The paired-pulse protocol was adopted to examine the recovery time course from the inactivation of Kv4.2 channels in the presence or absence of JZTX-X treatment (Fig. 7). The amplitude ratios of peak current responding to conditioning and test pulses were employed to evaluate the inhibition recovery, which was then plotted as a function of the interpulse interval. The inhibition recovery time courses were well matched to the singular exponential function, whereas the time constants were set at 130.2 ± 25.3 ms (n = 7, P<0.05) with JZTX-X treatment, and 195.3 ± 31.6 ms under controlled conditions (Fig. 7). The above findings suggested that JZTX-X remarkably accelerated the recovery of Kv4.2 channel inhibition.

### JZTX-X effects on the mechanical hypersensitivity and motor activity

**Figure 8.**
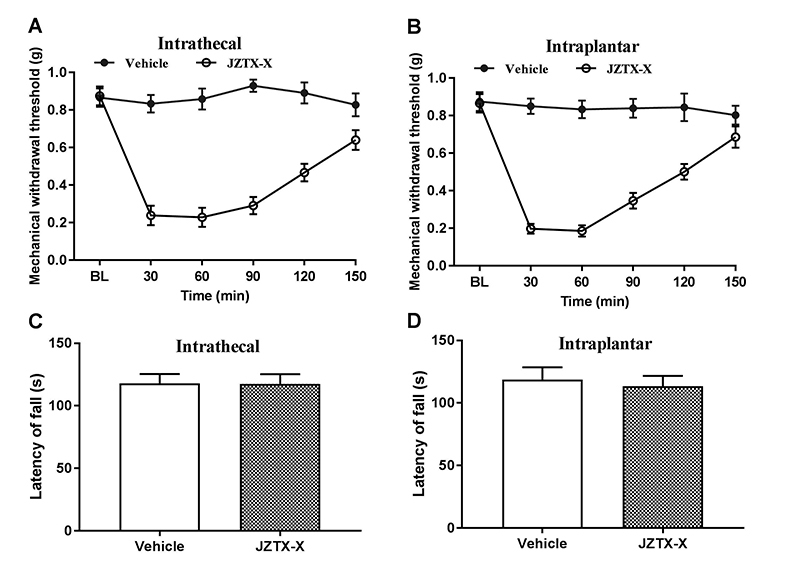
Effects of JZTX-X on the mechanical hypersensitivity and motor activity. **(** A **-** B **)** Effects of JZTX-X on mechanical hypersensitivity detected by von-Frey tests. Drugs were given to mice through intrathecal (A) or intraplantar (B) infusion. **(** C **-** D **)** Effects of JZTX-X on the motor activity detected by rotarod tests. Mice were given intrathecal (C) or intraplantar (D) infusion. Mice were put onto the rod at 30 min following JZTX-X treatment. Data were expressed as mean ± S.E. n = 8 rats for each group.

Subsequently, the present study explored the consequence of using JZTX-X on pain behavior in mice. According to our results, intrathecal or intraplantar injection of JZTX-X (40 nmol/kg) markedly decreased the baseline thresholds of tactile withdrawal in mice (n = 8, Fig. 8A and B). Notably, the reduced thresholds of paw withdrawal induced by JZTX-X lasted for at least 120 min after cessation of JZTX-X treatment. JZTX-X treatment did not induce spontaneous nociceptive behaviors (n=8). By contrast, vehicle treatment did not differ as to the thresholds of paw withdrawal, which is determined by the von Frey filaments (Fig. 8A-B). 

JZTX-X inhibited the Kv4 potassium currents and, consequentially the toxin potentially impacted the motor activity in animals. Therefore, the toxic effects on the motor coordination of mice were evaluated through the rotarod apparatus. Typically, mice that did not fall off the rotarod in the initial 300 s threshold were assigned a 300s latency. In vehicle-treated groups, mice stayed for 117.9 ± 7.6 s and 118.8 ± 9.8 s on the accelerating rotarod in intrathecal (Fig. 8C, n = 8) and intraplantar (Fig. 8D, n = 8) injection group, respectively. Specifically, the rotarod tests were conducted at 1 h following JZTX-X treatment. JZTX-X treatment (40 nmol/kg) made no difference to the motor coordination in mice, since they stayed on the drum for 117.5 ± 7.8 s (intrathecal injection group, Fig. 8C, n = 8) and 113.4 ± 8.3 s (intraplantar injection group, Fig. 8D, n = 8), respectively. These results were similar to those of the vehicle-treated mice, implying that JZTX-X did not affect the motor coordination at the dose that changed nociceptive responses.

## Discussion

The voltage-gated potassium channels are of crucial importance for controlling neuron and cardiocyte repolarization rates from excitable tissues [38]. JZTX-X, a new inhibitor of the Kv4 channels, was isolated from *C. jingzhao* venom, and its structure and functions were characterized. As suggested, JZTX-X had obvious effects on Kv4.2-4.3 channels, but they showed no effect on Kv1.1-1.3 or Kv2.1 channels in *Xenopus laevis* oocytes. Furthermore, this toxin inhibited the Kv4.2 and Kv4.3 channels in HEK293t cells in a concentration- and voltage-dependent manner. In contrast to the scorpion toxins that act by means of pore occlusion, many toxins from spiders play roles as the gating modifiers on ion channels through the voltage-dependent voltage-sensor trapping [39]. Our electrophysiological results suggested that the mechanism of JZTX-X in inhibiting Kv4 channels resembled those of HaTx1 and SGTx1 in suppressing Kv2 channels [40, 41], since they preferentially bound to the closed channel state depending on the voltage. According to our results, JZTX-X trapped the resting Kv4 channels, as proved by the findings below. First of all, JZTX-X inhibited the gating of Kv4.2 and Kv4.3 channels in a voltage-dependent manner. In addition, toxin resulted in the activation of Kv4.2 and Kv4.3 channels that shifted to higher depolarized voltages. Furthermore, channels binding to toxins were triggered through potent depolarization. JZTX-X completely inhibited the Kv4.2 and Kv4.3 channels at resting potential, even in the absence of the physical stimulus (Fig. 4). 

Our findings also revealed that the extent of JZTX-X-induced inhibition alteration was dependent on the depolarization potential. For instance, 500 nM JZTX-X almost totally suppressed the Kv4.2 current amplitude (~ 93%) induced at +20 mV voltage, but it was about ~ 46% at +130 mV, suggesting that there was toxin bound onto the channels, regardless of potent depolarization (Fig. 6). Thus, the Kv4.2 channels might have been activated even though JZTX-X was still bound onto those channels. Commonly, potent depolarization overcomes or reverses inhibitory action of spider-derived toxins. A similar finding was reported in relation to the effects of hanatoxins on the Kv2.1 channels [42], and the influences of heteropodatoxins (HpTx) and phrixotoxins (PaTx) on Kv4 channels [19]. It has been widely indicated that these peptide toxins inhibit the voltage-gated potassium currents through trapping at least one voltage sensor within a closed configuration [19, 20, 42]. Thus, JZTX-X possibly inhibited the activation of Kv4.2 and Kv4.3 channels by a similar mechanism, but more rigorous functional or structural studies should be conducted for verification.

The Kv4.2 channel has been identified as a potent factor contributing to the A-type currents within the dorsal horn neurons, which are also in the crucial position for regulating pain processing [43]. Furthermore, the Kv4.3 channel can be detected within the somata of a neuronal subset in non-peptidergic nociceptive dorsal root ganglion (DRG), which plays a critical role in the control over neuron excitability, while its down-regulated level in the neurons sensing pain can boost pain sensation [44, 45]. Because JZTX-X inhibited the activation of both Kv4.2 and Kv4.3 channels in a closed state, it was not surprising that JZTX-X induced long-lasting mechanical hyperalgesia in mice. A spider may bite when it is threatened by restraint or by touching. One of the most common functions of spider venom is to defend the spider against predators or potential enemies [46]. Some clinical studies suggest that most venomous bites by spiders induce obvious local effects, such as pain and a red mark surrounding those bite sites [47]. Understanding the mechanism of venom-induced pain at the molecular level may help the researchers to develop a treatment for bitten patients, and to establish pharmacological approaches to reducing their sufferings.

**Figure 9.**
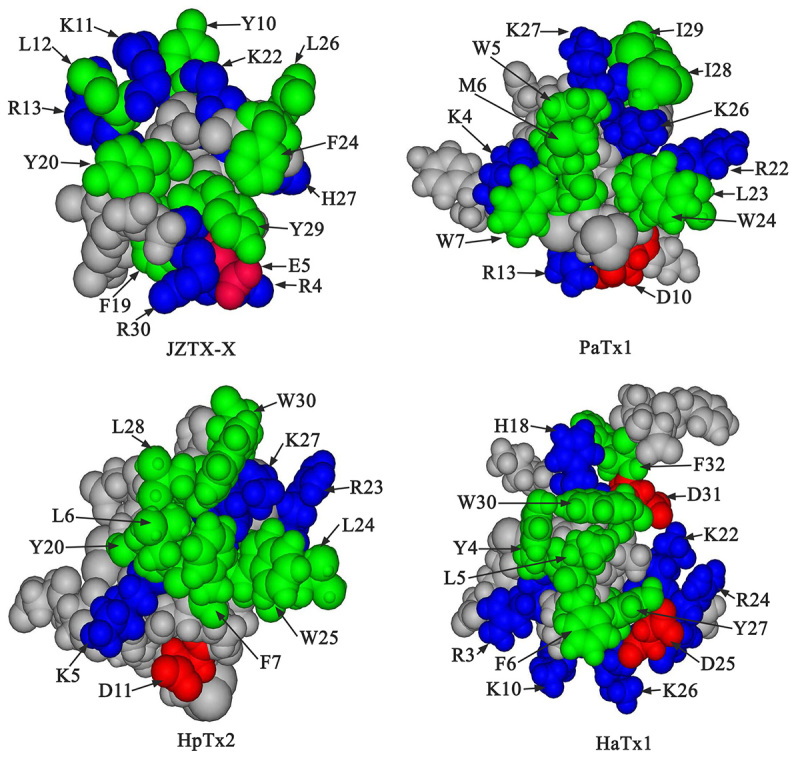
3D structures of JZTX-X, PaTx1, HaTx1, and HpTx2: The JZTX-X models were acquired through homology modeling according to ω-GsTx SIA structure identified by NMR (PDB code: 1KOZ). The DS ViewerPro was utilized to produce CPK models. Those critical amino-acid residues predicted for PaTx1 to inhibit the potassium channels were marked with different colors. Candidate functional residues for the remaining three toxins were also denoted. Green, hydrophobic (Tyr, Ile, Met, Leu, Trp, Phe); Blue, basic (Lys, His, Arg); Red, acidic (Glu, Asp).

Moreover, JZTX-X, which belongs to the ICK peptide family, usually emerges in spiders and marine snails as suggested by our results [39]. Numerous spider peptides binding to the voltage-gated sodium, calcium or potassium channels possess identical structural configuration (the ICK motif that contains the tight hydrophobic core bonded by disulfides, with only certain short loops in this core) [48]. To shed more light on the association between JZTX-X and Kv4 potassium channels, a 3D JZTX-X model was established (As shown in Fig. 9) based on NMR structures of omega-grammotoxin SIA. The structure of JZTX-X is compared with those of the gating-modifying toxins (such as HaTx1, HpTx2, as well as PaTx1), which indicates that the latter ones possess a potent hydrophobic moment. The face inserting into the membrane is nearly completely hydrophobic, and binds to those channel voltage sensors. Furthermore, those negatively- or positively-charged residues have surrounded the hydrophobic patch [49-51]. Based on the above-mentioned previous findings, the hydrophobic patch surface motif which is encompassed with the positively charged residues onto the gating-modifying toxins, possibly accounts for the binding onto the voltage-gated ion channels. 

## Conclusions

In conclusion, our findings suggest that JZTX-X inhibits the activation of both Kv4.2 and Kv4.3 channels under a resting state in a concentration- and voltage-dependent manner. Such novel findings further elucidate the molecular mechanism of spider-venom-induced lengthened pain sensation. The function of JZTX-X indicates it as a suitable new tool for investigating Kv4.2 and Kv4.3 channels, which also suggests that targeting at Kv4.2 and Kv4.3 channels may constitute a favorable strategy to treat neuropathic hyperalgesia.

### Abbreviations

DRG: dorsal root ganglion; HPLC: high-performance liquid chromatography; HpTx: heteropodatoxin; IC_50_: median inhibitory concentration; ICK: inhibitor cysteine knot; JZTX: jingzhaotoxin; JZTX-X: jingzhaotoxin-X; PaTx: phrixotoxin; Kv channel: voltage-gated potassium channel; MALDI-TOF: matrix-assisted laser desorption/ionization time-of-flight.
